# Intrauterine bony fragments - An unexpected finding in the hysterectomy specimen

**DOI:** 10.4322/acr.2020.182

**Published:** 2020-09-02

**Authors:** Madhu Chaturvedi, Ankita Shende

**Affiliations:** a Lokmanya Tilak Municipal Medical College and Lokmanya Tilak Municipal General Hospital (LTMGH), Pathology Department. Mumbai, Maharashtra, India

**Keywords:** Abortion, Incomplete, Dilatation and Curettage, Hysteroscopy, Ultrasonography

## Abstract

Intrauterine bony fragments (IUBF) are an unusual finding in hysterectomy specimen received in a histopathology laboratory. Females harboring IUBF may present non-specific symptoms like vaginal bleeding, leukorrhea, chronic pelvic pain, and secondary infertility. Herein we report the case of a 35-year-old female who presented vaginal discharge and bleeding for two years, since when she had an abortion. Later, hysterectomy specimen revealed bone pieces in the uterine cavity.

## CASE REPORT

**Figure 1:**
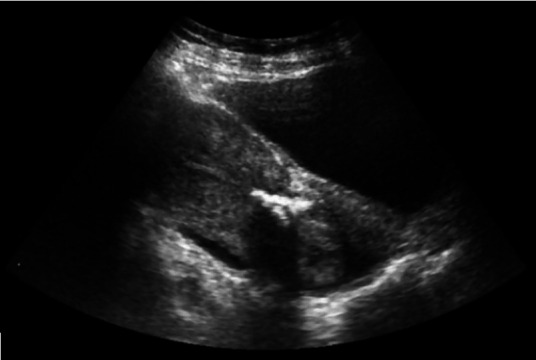
Pelvic Ultrasound showing heterogenous & hyperechoic content with multiple calcific foci within the endometrial cavity.

We received an endometrial biopsy of a 35-year-old female with a parity index of G4P3L3A1. The patient complained of polymenorrhea and leucorrhea for 2 years. Her medical history included an abortion two years ago. Her first delivery was by lower segment cesarean section because of a breech presentation, and the last delivery was full-term normal delivery seven years ago. A recent pelvic ultrasonography depicted heterogeneous and hyperechoic contents within the uterine cavity, along with multiple calcific deposits suggestive of osseous metaplasia of the endometrium (Figure 1).

**Figure 2:**
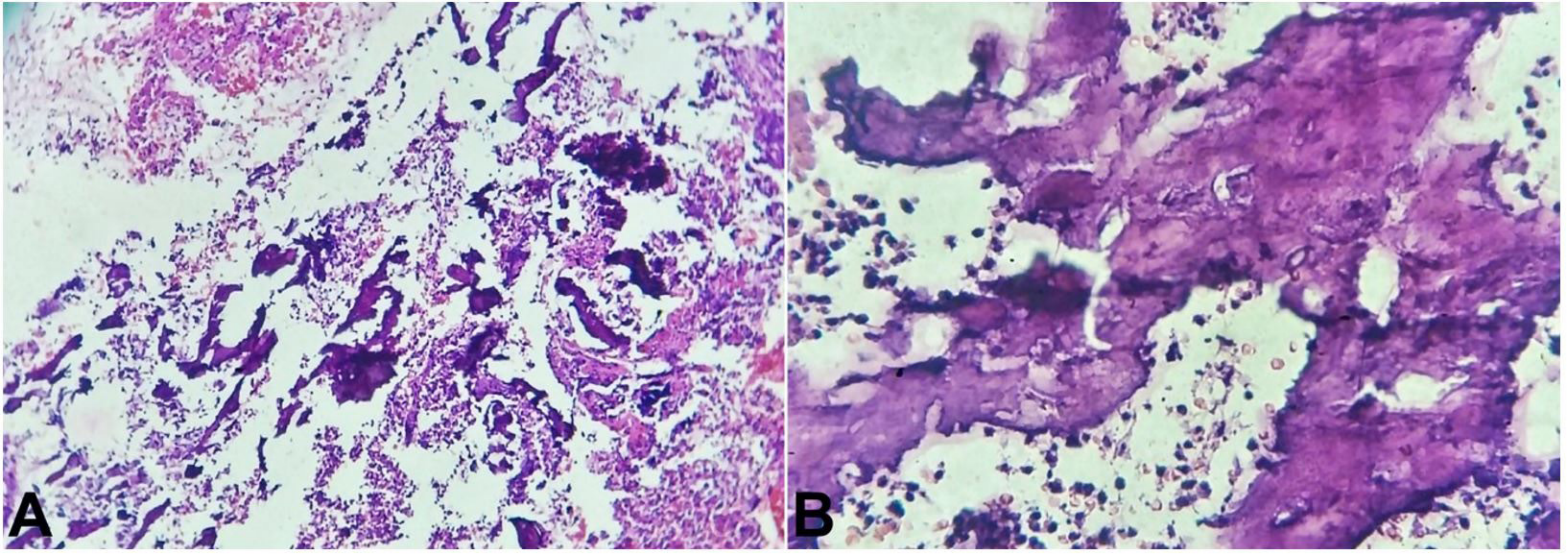
Photomicrograph of the endometrial biopsy (**A** and **B**) shows stroma and bone tissue and calcification, (H& E, 40X).

The histopathology of the endometrial biopsy showed an endometrium with bony remnants (Figure 2A). The bony remnants showed mature degenerated osteocytes, bone tissue, and calcification (Figure 2B).

**Figure 3:**
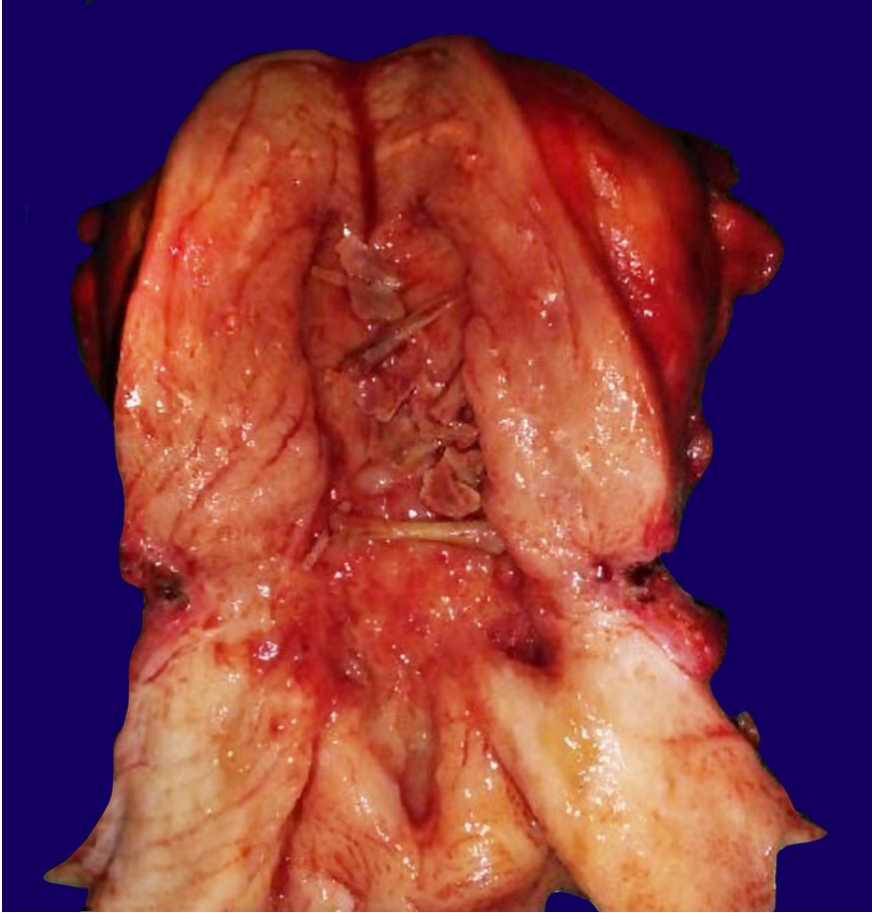
Gross view of the surgical specimen showing a simple hysterectomy with retained fetal bones in the endometrial cavity.

The endometrial stroma showed a florid chronic inflammation predominantly composed of plasma cells and a few lymphocytes. Therefore, the report of chronic endometritis with osseous metaplasia was rendered. After a few days, we received the same patient’s hysterectomy specimen, which showed intrauterine bones in the endometrial cavity (Figure 3).

## DISCUSSION

Intrauterine bone fragments are an unexpected finding in a hysterectomy specimen received in the Pathology Department for morphological examination. The clinical presentation of IUBF is non-specific and includes vaginal discharge, bleeding, leucorrhoea, secondary infertility, chronic pelvic pain, and dysmennorrhea.^1^ Makris et al.^2^ reported the incidence of 0.15% in 2000 diagnostic hysteroscopies, while this incidence was 0.26% in 1002 hysteroscopies in the study of Okohue,^3^ in Nigeria (where abortion laws are highly restrictive). Gainder S et al.^4^ reported the incidence of IUBF of 0.28% in144 women with infertility. These women were diagnosed to have retained fetal bones on the pelvic sonogram, which was later confirmed and removed by hysteroscopy.

In all these studies and many other single case reports,^5^ ^-^ ^7^ most of the females presented with infertility and had a history of prior abortion. Other cases presented with pelvic pain, vaginal discharge and, abnormal uterine bleeding.^8^ ^-^ ^10^

In their global, comprehensive, and systematic review on IUBF, Khan et al.^11^ reviewed 293 cases reported between the years 1928 and 2013. In this study, the mean ± SD age at presentation was 32.7 ± 8.9. Approximately 88% of patients had at least one prior surgical uterine evacuation related to pregnancy termination or loss at a median gestational age of 14 weeks (range of 4-41 weeks). The most common presenting symptom was infertility (56.2%) followed by irregular bleeding (19.8%), and vaginal discharge was reported in 6.4% of cases. Out of the 293 patients, 7.4% were from the Indian subcontinent, and of 155 reviewed articles, only 5.8% were reported from India. The main diagnostic modality was ultrasonography (65.4%), and the treatment modality was hysteroscopic excision (67.4%). In contrast, non-hysteroscopic approach, namely (i) biopsy, (ii) forceps, (iii) dilation and curettage, and (iv) hysterectomy occurred in 32.2% patients. Hysterectomy was performed in 12 cases. All these women were perimenopausal and completed childbearing. Symptom resolution was reported in 52 of 64 (81.3%) patients who did not present with infertility, while failure to resolve the symptom was reported in 3 (4.7%), and 9 patients (14.1%) were lost to follow up. In 188 cases (64.2%), the information of the willingness or not to get pregnant was available; out of them, 124 women (66.0%) attempted to get pregnant, and 90 (72.6%) achieved pregnancy.

The finding of bone within the uterus or endometrium has been described as ossification of the endometrium, osseous metaplasia of endometrium, ectopic intrauterine bone, and heterotopic intrauterine bone. Some pathogenic theories of bone in the endometrium have been proposed in the literature. Heterotopia, dystrophic calcification, ossification of post-abortive endometritis, metastatic calcification, and prolonged estrogen therapy after abortion, genital tuberculosis, and retained fetal bone are the commonly proposed theories. The two most accepted mechanisms involve either dystrophic calcification of residual ovular tissue after abortion or termination of pregnancy or can be secondary to the transformation of endometrial stromal cells into osteoblasts following chronic endometritis.^12^ ^,^ ^13^

The main sonographic finding of endometrial osseous metaplasia is the presence of a strongly echogenic endometrial plate. Other possible diagnoses include the presence of foreign bodies, Asherman’s syndrome, calcified submucosal fibrosis, and Mullerian tumor. However, the diagnosis of endometrial osseous metaplasia should be taken into consideration in cases where strongly echogenic endometrial plates are detected in patients with a history of miscarriage and chronic endometritis.^13^

In the present case, the correlation between the history of antecedent abortion and the pelvic sonogram showed hyperechoic contents allowed the diagnosis of osseous metaplasia. Later, it was confirmed by histopathology of endometrium collected by means of dilatation curettage and presence of bone pieces in the endometrial cavity in hysterectomy specimen.

Published literature shows that it is treatable by non-invasive hysteroscopy.^3^ ^,^ ^6^ ^,^ ^13^ In the present case, the hysterectomy was performed because the female had completed childbearing.

## CONCLUSION

Intrauterine bone fragments are an unusual entity. It becomes an odd cause of morbidity in females. The surgical pathologist should be aware of this condition. It is a combined clinical, pathological, and radiological diagnosis. On histopathology, whenever an endometrial biopsy is received, and endometrial osseous metaplasia is suspected, it is prudent to ask for a detailed history including past obstetric history, especially abortion (legal or illegal) and post-abortion ultrasonography report.
